# Association of Tissue Expression of LAG-3 and TIM-3 with Clinical Features in Ovarian Cancer

**DOI:** 10.3390/ijms26135996

**Published:** 2025-06-22

**Authors:** Dominika Borzyszkowska, Mateusz Kozłowski, Anna Golara, Katarzyna Piotrowska, Agnieszka Brodowska, Jacek Brodowski, Iwona Bojar, Agnieszka Kempinska-Podhorodecka, Aneta Cymbaluk-Płoska

**Affiliations:** 1 Department of Reconstructive Surgery and Gynecological Oncology, Pomeranian Medical University, Al. Powstańców Wielkopolskich 72, 70-111 Szczecin, Poland; domi98nikab@gmail.com (D.B.); mateusz.kozlowski@pum.edu.pl (M.K.);; 2 Department of Physiology, Pomeranian Medical University, Al. Powstańców Wielkopolskich 72, 70-111 Szczecin, Poland; katarzyna.piotrowska@pum.edu.pl; 3 Department of Gynecology, Endocrinology and Gynecologic Oncology, Pomeranian Medical University, ul. Unii Lubelskiej 1, 71-252 Szczecin, Poland; 4 Primary Care Department, Pomeranian Medical University, Żołnierska 48, 71-210 Szczecin, Poland; 5 Department of Women’s Health, Institute of Rural Health, 20-093 Lublin, Poland; 6 Department of Medical Biology, Pomeranian Medical University, 70-111 Szczecin, Poland; agnieszka.kempinska.podhorodecka@pum.edu.pl

**Keywords:** immune biomarker, ovarian cancer, HGSOC, marker, TIM-3, LAG-3, expression

## Abstract

One of the most prevalent types of cancer among women is ovarian cancer. The search for ovarian cancer markers is constantly ongoing. Evaluation of LAG-3 and TIM-3 protein expression in ovarian cancer tissue and its role in distinguishing the clinical signs stated were the objectives of this study. Methods: A total of 58 ovarian cancer patients were recruited for this study. The cohort was split into two groups: one for high-grade serous ovarian cancer (HGSOC) and another for ovarian cancer that was not HGSOC (non-HGSOC). LAG-3 and TIM-3 protein expression in ovarian cancer tissue samples was evaluated by immunohistochemistry. StatView 5.0 software (Carry, NC, USA) was used for all statistical analyses. Both LAG-3 and TIM-3 proteins mostly showed positive, moderately positive, or strongly positive expression. This study shows that LAG-3 could be a marker associated with BMI in the non-HGSOC group. TIM-3 may be a marker associated with age in a group of all ovarian cancers. LAG-3 expression is associated with TIM-3 expression in the total cohort and the HGSOC and non-HGSOC groups.

## 1. Introduction

Ovarian cancer (OC) is the eighth most common cancer in women, making it an important focus in contemporary oncology [1]. The American Cancer Society’s Cancer Statistics Center estimates that in 2024 in the United States, about 19,680 women will receive a new diagnosis of OC, and about 12,740 women will die from it [2]. There are no specific symptoms of OC in its early stages; progression to advanced stages is often rapid. As a result, more than 70% of patients are diagnosed at an advanced stage of the disease [3]. Currently, there is no effective screening method for OC. Commonly used tests, such as Cancer Antigen 125 (CA-125) or Human Epididymis Protein 4 (HE4) levels in the blood, are of limited value due to low specificity, and their levels increase in other cancers and other conditions [4,5]. Multicenter, long-term randomized trials have not shown a significant reduction in mortality in patients undergoing screening [6,7]. Therefore, in addition to efforts aimed at improving early diagnosis, it is crucial to explore and develop new therapeutic strategies. Recent advances in oncology are increasingly moving toward personalized medicine. Furthermore, intensive research on cancer biomarkers has opened new avenues for individualized treatment approaches [8]. Inhibitors targeting Programmed Death-1 (PD-1), Programmed Death-Ligand 1 (PD-L1), and Cytotoxic T-Lymphocyte-Associated Protein 4 (CTLA-4) have recently contributed to the development of novel clinical anticancer strategies [9,10]. However, many OC patients are either inherently resistant to these inhibitors or eventually develop resistance, highlighting the need to identify alternative targets [10]. Our team conducted a literature review on the role of T-cell immunoglobulin and mucin domain-containing protein 3 (TIM-3) and Lymphocyte Activation Gene 3 (LAG-3) in the tumor microenvironment and immunotherapy of ovarian cancer [11]. Both proteins appear to be promising biomarkers due to their differential expression and their role as immune checkpoint molecules, potentially serving as effective targets for personalized antibody-based cancer therapies. TIM-3 protein as the inhibitory checkpoint receptor has previously drawn attention due to its expression on activated CD4+ and CD8+ T cells [12]. The protein was initially studied for its involvement in allergic reactions and autoimmune diseases due to the location of the TIM protein gene on chromosomes at sites associated with allergies and autoimmunity [13]. Currently, its expression has been studied in gastric cancer tissues [14] and in non-small cell lung cancer, and the literature analyzes it not only as a prognostic marker but also sees it as a target with great therapeutic potential if a patient develops resistance to anti-PD1 therapy [15,16]. As for the study of TIM-3 in gynecologic cancers, it showed that PD-L1 and TIM-3 are prognostically significant biomarkers of active and suppressed immune responses against high-grade serous ovarian cancer, and this study demonstrated that TIM-3 dictates the functional orientation of immune infiltration in ovarian cancer [17]. Another study showed that genetic variants and expression of the TIM-3 gene are associated with clinical prognosis in patients with epithelial ovarian cancer [18].

Like TIM-3, LAG-3 is an immune checkpoint that is expressed on the surface of many immune cells [19]. T cell exhaustion can result from the co-expression of LAG-3 with other immunological checkpoints [20]. These exhausted T cells are less cytotoxic and less able to generate cytokines, which affects their capacity to identify and eliminate tumor cells [21]. LAG-3 has been studied in various gynecological cancers: endometrioid endometrial cancer, high-grade serous ovarian cancer (HGSOC), and clear cell ovarian cancer [22,23,24]. A study of LAG-3 as a potential immunotherapeutic target for microsatellite-stable, PD-L1+ endometrioid endometrial cancer has substantiated that LAG-3 may be a potential target for immunotherapy in this cancer [22]. In patients with high-grade serous ovarian cancer (HGSOC), it was shown that circulating LAG-3 is associated with better survival in this cancer [23]. In clear cell ovarian cancer, increased LAG-3 expression in tumor-infiltrating lymphocytes was associated with FIGO stages III and IV and in cases of cancer recurrence [24].

The purpose of this study was to evaluate LAG-3 and TIM-3 protein expression in ovarian cancer tissue and its association with clinical features.

## 2. Results

### 2.1. Group Characteristics and Evaluation of Tissue Expression

**Figure 1:**
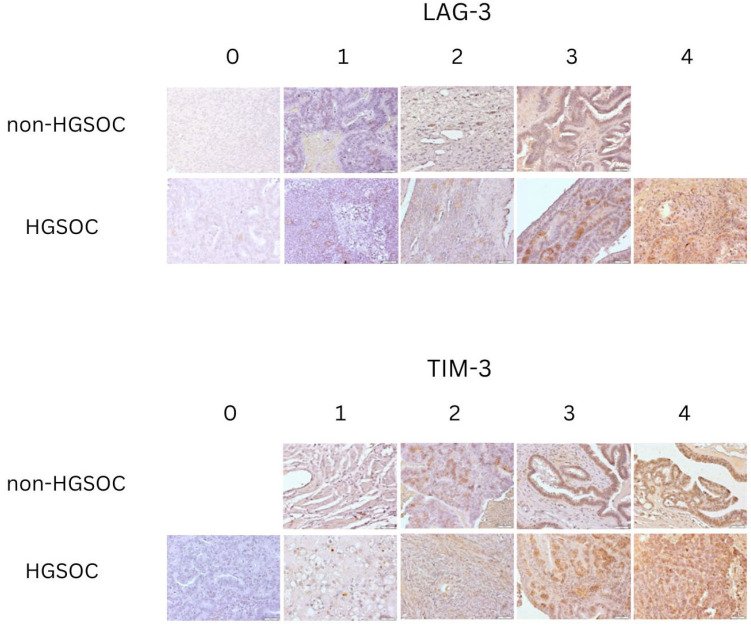
IHC evaluation of LAG-3 and TIM-3: study and control group samples: 0—negative reaction; 1—weakly positive reaction; 2—positive reaction; 3—moderately positive reaction; 4—strongly positive reaction. Objective magnification ×20, scale bars 50 µm.

The expression of LAG-3 and TIM-3 was analyzed in both the non-HGSOC group and the HGSOC group. In each group, expression intensity was classified into the following categories: 0—negative reaction, 1—weakly positive reaction, 2—positive reaction, 3—moderately positive reaction, and 4—strongly positive reaction. The immunohistochemical expression is illustrated in Figure 1.

A total of 11% showed negative expression (intensity: 0) of LAG-3 in the low-grade group. A total of 28% showed weakly positive expression (intensity: 1). A total of 39% showed positive expression (intensity: 2), making it the most common category. A total of 22% showed moderate expression (intensity: 3). There were no cases with strong LAG-3 expression (intensity: 4) in the low-grade group.

A total of 30% showed no expression (intensity: 0) of LAG-3 in the high-grade group. A total of 20% showed weakly positive expression (intensity: 1). Positive expression (intensity: 2) was the most commonly observed expression in the high-grade group and accounted for 35%. Moderate expression (intensity: 3) of LAG-3 was observed in 15% of cases. There were no cases with strong LAG-3 expression (intensity: 4) in the high-grade group.

There were no cases with negative expression (intensity: 0) of TIM-3 in the low-grade group. A total of 17% showed weakly positive expression (intensity: 1), and 22% showed positive expression (intensity: 2). A total of 44% showed moderate expression (intensity: 3), making it the most common category. A total of 17% showed strong expression (intensity: 4) of TIM-3.

A total of 18% showed no expression (intensity: 0) of TIM-3 in the high-grade group. A total of 20% showed weakly positive expression (intensity: 1) of TIM-3. A total of 27% showed positive expression (intensity: 2). The most commonly observed category was moderate expression (intensity: 3), present in 30% of cases. A total of 5% showed strong expression (intensity: 4).

To further explore potential associations, clinical–demographical characteristics were analyzed. Considering age and BMI as continuous characteristics, there was a significant difference in age between the HGSOC and non-HGSOC groups (*p* = 0.005). The difference in BMI between the two groups was not significant. The clinical–demographical characteristics tested were then divided into two subgroups and compared between HGSOC and non-HGSOC groups. Details of the group characteristics are shown in Table 1.

### 2.2. Comparison of Tissue Expression of LAG-3 Between Non-HGSOC and HGSOC

In the group of 0–1 LAG-3 protein expression intensity, clinical features were compared between HGSOC and non-HGSOC. There was a significant difference in grade (*p* = 0.0002) and histological type (*p* = 0.0008). For menopausal status, FIGO, BMI, and age differences were not significant. Details are shown in Table 2.

In the group of 2-4 LAG-3 protein expression intensity, clinical features were compared between HGSOC and non-HGSOC. There was a significant difference in grade (*p* = 0.0001), histological type (*p* = 0.0007), menopausal status (*p* = 0.005), FIGO stage (*p* = 0.001), and BMI (*p* = 0.04). Differences in age were not significant. Details are shown in Table 3.

### 2.3. Comparison of Tissue Expression of TIM-3 Between Non-HGSOC and HGSOC

In the group of 0-1 TIM-3 protein expression intensity, clinical features were compared between HGSOC and non-HGSOC. There was a significant difference in grade (*p* = 0.007), histologic type (*p* = 0.007), and FIGO stage (*p* = 0.04). Differences in menopausal status, BMI, and age were not significant. Details are shown in Table 4.

In the group of 2-4 TIM-3 protein expression intensity, clinical features were compared between HGSOC and non-HGSOC. There was a significant difference in grade (*p* = 0.0001), histologic type (*p* = 0.0001), and FIGO stage (*p* = 0.003). Differences in menopausal status, BMI, and age were not significant. Details are shown in Table 5.

### 2.4. Association of LAG-3 and TIM-3 Protein Expression with Clinical Features

Correlations between LAG-3 and TIM-3 protein expression and the described clinical features were evaluated. In the total ovarian cancer cohort (HGSOC and non-HGSOC), there was a weak negative significant correlation between TIM-3 and age (r = −0.291) and a moderately positive significant correlation between TIM-3 and LAG-3 (r = 0.609). For the HGSOC group, there was a moderately positive significant relationship between LAG-3 and TIM-3 (r = 0.535). For the non-HGSOC group, there was a fairly strong positive significant relationship between LAG-3 and TIM-3 (r = 0.710) and a moderately positive significant relationship between LAG-3 and BMI (r = 0.405). Detailed data are shown in Table 6, Table 7 and Table 8.

## 3. Discussion

One of the primary reasons for the immune system’s failure to control tumor growth is the suppression of immune reactivity caused by increased expression of co-inhibitory receptors, which may play a key role in shaping future therapeutic strategies. Rådestad et al. used flow cytometry to analyze lymphocytes extracted from blood, ascites, and tumor tissue of patients with advanced ovarian cancer (*n* = 35), identifying markers involved in T cell function and regulation. The co-inhibitory receptors LAG-3, PD-1, and TIM-3 were expressed by significantly higher proportions of CD4+ and CD8+ T lymphocytes in tumor tissue and ascites compared to peripheral blood. PD-1 and TIM-3 were the most often found combination, and co-expression was primarily seen among intratumoral CD8+ T cells. A higher proportion of CD8+ T cells lacking PD-1, TIM-3, or LAG-3 expression was associated with improved overall survival, based on the correlation with clinical outcomes [25]. The clinical prognosis of individuals with epithelial ovarian cancer is correlated with genetic variations and TIM-3 gene expression. When compared to controls, patients’ peripheral blood CD4+ and CD8+ T lymphocytes have noticeably higher levels of TIM-3 expression. Compared to patients with low expression, those with high TIM-3 expression have greater tumor grades and clinical stages. TIM-3 may therefore be a viable therapeutic target for EOC and may have a significant impact on the onset and course of EOC [18,26].

Since TIM-3 has been implicated in the development of several ovarian cancer subtypes, it may represent a viable therapeutic target for ovarian cancer treatment [27]. Using a retrospective cohort of 80 chemotherapy-naive HGSC patients, Fucikova et al. investigated the relationship between prognosis and the functional orientation of the tumor microenvironment, including PD-L1 expression and infiltration by CD8+ T cells, CD20+ B cells, DC-LAMP+ dendritic cells, as well as PD-1+, CTLA-4+, LAG-3+, and TIM-3+ cells. In a second prospective cohort of recently resected HGSC samples, transcriptomic and functional analyses were conducted alongside immunohistochemistry. PD-1+TIM-3+CD8+ T cells were associated with poor prognosis and exhibited features of functional exhaustion. The prognostic value of intratumoral CD8+ T cell density was enhanced when combined with PD-L1 levels and the extent of TIM-3+ cell infiltration.

Thus, TIM-3 and PD-L1 may serve as prognostically significant indicators, reflecting a repressed and an active immune response against HGSC, respectively [17]. Higher levels of TIM-3 expression on regulatory T cells (Tregs) are associated with poorer progression-free survival. This association is observed in both BRCA wild-type (BRCAwt) and germline BRCA-mutated (gBRCAm) patients. Therefore, blocking TIM-3 signaling pathways could represent a promising strategy for limiting tumor progression [28]. Immune checkpoint molecules such as TIM-3 and LAG-3 are associated with poor prognosis in ovarian cancer. Moreover, there is a strong correlation between immune cell infiltration and the expression of PD-1, CTLA-4, TIM-3, and LAG-3 in the ovarian cancer microenvironment [29].

Durand et al. examined the expression of four immune checkpoint molecules—TIM-3, IDO (indoleamine 2,3-dioxygenase), LAG-3, and PD-L1—in ovarian cancer (OvCa) cells of various histologic subtypes, including high-grade serous, low-grade serous, mixed, poorly differentiated, endometrioid, clear cell, and mucinous carcinoma. Compared to PD-L1, IDO, and LAG-3, TIM-3 showed markedly higher expression levels in ovarian cancer tumors. In over 75% of the samples, TIM-3 was the most prevalent and abundant co-inhibitory molecule. More than 50% of ovarian cancer cases expressed two, three, or even all four of these checkpoint molecules. When considering only high-grade serous samples, the expression pattern of these four biomarkers closely resembled that observed in the overall cohort [30]. Malignant ascites lymphocytes (MALs) and Vδ1 peripheral blood lymphocytes (PBLs) from ovarian cancer patients exhibited the highest levels of TIM-3 expression. In these patients, the expression of TIM-3, TIGIT, PD-1, and CD39 on Vδ1 cells varied among PBLs, MALs, and tumor-infiltrating lymphocytes (TILs), depending on the tumor microenvironment [31].

Our study also demonstrated that differences in TIM-3 protein expression levels—both lower and higher—were associated with variations in tumor severity, histological subtype, and FIGO stage. Thus, TIM-3 may serve as a potential biomarker correlated with tumor stage, histological subtype, menopausal status, FIGO stage, and body mass index (BMI) across the spectrum of ovarian cancer cases.

Research on immunotherapeutic vaccines that target TIM-3 is still lacking. A particular shRNA that targets the human TIM-3 gene was created and placed into a lentiviral vector containing a transgenic MSLN-CAR-T construct in a work by Jafarzadeh et al. The cytotoxic activity, cytokine generation, and proliferative potential of MSLN-CAR-T cells were all markedly enhanced by TIM-3 knockdown, which also considerably decreased its expression in these cells. By allowing tumor-infiltrating CAR-T cells to multiply and perform well, targeted TIM-3 knockdown reduces TIM-3-mediated immune suppression [32]. In high-grade EOC, CAR-T treatment has shown a long-lasting clinical response. Clinical trials using anti-mesothelin CAR T cells, or mesoCAR T cells, are being conducted in a number of cancer types, including EOC. When meso-CAR T cells were generated for advanced human epithelial OC, high expression of inhibitory receptors such as PD1, TIM-3, and A2aR was noted. This suggests that pre-activation of T cells may not be necessary if CAR T cell production techniques are optimized [33].

The expression of various immune components is frequently compared to LAG-3 expression in ovarian cancer. Analyses are conducted on tumor-infiltrating lymphocytes (TILs), as well as lymphocytes derived from peripheral blood and ascites. LAG-3 expression is evaluated in the context of diverse clinical settings and tumor microenvironmental factors. Huang et al. investigated LAG-3 expression in both human samples and a mouse model. Tumor-associated lymphocytes (TALs) from ovarian cancer patients confirmed that both CD8+ and CD4+ T cells exhibited elevated levels of LAG-3 and PD-1. Furthermore, a substantial population of PD-1^+^TIM-3^+^CD8^+^ and CD4^+^ T cells was identified in human TALs. In the mouse model, inhibition of PD-1–LAG-3 or PD-1–CTLA-4 signaling pathways was shown to reduce tumor growth. The authors also reported that, when the PD-1 pathway was completely inhibited, triple blockade of PD-1, CTLA-4, and LAG-3 was more effective than dual blockade [34]. In another murine study, TILs also demonstrated elevated expression of LAG-3 and its co-expression with PD-1. Simultaneous blockade of both molecules improved antitumor immunity by decreasing the frequency of regulatory T cells (Tregs) in the tumor microenvironment and increasing the proportion and activity of CD8^+^ effector T cells [35]. The enrichment of LAG-3^+^CD8^+^ T cells within the PD-1^+^ T cell subset further supports the notion of co-expression of these immune checkpoints [36]. Interestingly, LAG-3 expression has also been correlated with improved survival in ovarian cancer patients [37]. Ma et al. used bioinformatics analysis to investigate LAG-3 expression in subtypes of ovarian serous cystadenocarcinoma—early-onset (EOOSC) and late-onset (LOOSC). Their findings indicated that a subset of EOOSC patients exhibited lower LAG-3 expression, while higher LAG-3 levels were associated with longer overall survival [38]. We also showed in our study that both patients with lower and higher LAG-3 protein expression showed differences in grade and histological type. Patients with higher LAG-3 protein expression showed differences in menopausal status, FIGO stage, and BMI, which was not observed in patients with lower expression.

The obtained results indicate that the expression of LAG-3 and TIM-3 proteins may be associated with specific clinicopathological features of ovarian cancer, such as histological type, FIGO stage, menopausal status, BMI, and patient age. These relationships may be important for the future differentiation of ovarian cancer subtypes and for identifying patients with a worse prognosis or a different course of the disease. The observed relationship of LAG-3 and TIM-3 expression with clinical parameters suggests that both proteins may act not only as prognostic but also predictive biomarkers. Moreover, their simultaneous expression in various subtypes of ovarian cancer indicates the possibility of common participation in the mechanisms of immunosuppression, which may be important from the point of view of immunotherapy. LAG-3 and TIM-3 are already the subject of clinical trials in the treatment of other cancers (e.g., melanoma, lung cancer, breast cancer), where their blockade leads to the activation of the immune response and increased therapy effectiveness. From a clinical point of view, these results open the prospect of using LAG-3 and TIM-3 inhibitors as adjunctive therapies in the treatment of ovarian cancer, potentially in combination with PD-1/PD-L1 and CTLA-4 inhibitors, as well as with currently used drugs such as bevacizumab or PARP inhibitors. This may be particularly important in the case of patients with cancer that is resistant to standard treatment or in the relapse phase of the disease. However, it should be emphasized that the presented results are preliminary and require further validation in studies involving larger cohorts of patients and in experimental models. Only such analyses will allow for a full assessment of the translational potential of the tested biomarkers and their actual application in clinical practice.

This study has several important limitations. First, the relatively small number of patients limits the generalizability of the results to the broader population of ovarian cancer patients. Secondly, separating the cohort into HGSOC and non-HGSOC groups further reduced the size of individual subgroups. Thirdly, the non-HGSOC group is heterogeneous and includes several histological subtypes of ovarian cancer with varying grades, serving as the control group in this study. Additionally, LAG-3 and TIM-3 protein expression was assessed only by immunohistochemistry, which may require further validation using quantitative methods.

## 4. Materials and Methods

### 4.1. Study Design

This study presented here was a retrospective study. Patients with diagnosed and histologically confirmed ovarian cancer were qualified for this study. The entire group was categorized into two groups: one for high-grade serous ovarian cancer (HGSOC), which was the study group, and another for non-high-grade serous ovarian cancer (non-HGSOC). Furthermore, the group was separated into smaller groups based on the following criteria: FIGO (International Federation of Gynecology and Obstetrics) histological type, grading, and clinical stage. The study material was formalin-fixed paraffin-embedded (FFPE) ovarian cancer tissue.

This study was conducted in accordance with the Declaration of Helsinki and approved by the Bioethics Committee of the Pomeranian Medical University in Szczecin (protocol code KB-006/49/2022 on 16 November 2022).

The histological diagnosis of ovarian cancer was used for this study’s inclusion criterion. The criterion for division into the study and control groups was histological type and grading. Exclusion criteria included pelvic inflammatory illnesses, uncompensated chronic disease, treatment history for another malignancy, absence of patient consent, inadequate patient data, and histological diagnosis of uterine malignancy other than cancer. In the end, 58 patients met this study’s eligibility requirements. At the beginning of this study, the patients’ body mass indices (BMIs) were calculated based on their height and weight. The formula used to determine the BMI was BMI = weight (kg)/height^2^ (m^2^). The patients were classified into two subgroups based on the results: those with BMI < 25 and those with BMI ≥ 25. Additionally, patients were separated into premenopausal and postmenopausal subgroups based on their menopausal status.

### 4.2. Immunohistochemical (IHC) Expression of LAG-3 and TIM-3 in Ovarian Cancer Tissue Samples

Sections of ovarian cancer samples (4 μm thick) were hydrated, and heat epitope retrieval was performed in a microwave oven in citrate buffer pH = 6 (Dako Retrieval Solution, Dako Denmark, Glostrup, Denmark). After cooling to room temperature (RT), the activity of peroxidase was blocked with BLOXALL Endogenous Enzyme Blocking Solution (ImmPRESS^®^ HRP Universal (Horse Anti-Mouse/Rabbit IgG) PLUS Polymer Kit, Peroxidase, Vector Laboratories, Newark, CA, USA), washed twice with PBS, and further incubated with 2.5% Normal Horse Serum (ImmPRESS^®^ HRP Universal PLUS Polymer Kit, Peroxidase, Vector Laboratories, Newark, CA, USA). Further, slides were incubated with primary monoclonal antibodies: mouse anti-LAG-3 (Novous Bio, BioTechne, Minneapolis, MN, USA) and mouse monoclonal TIM-3 (Invitrogen, ThermoFisher Scientific, Waltham, MA, USA) for 1 h at RT. After a double wash in PBS, slides were incubated with ImmPRESS Universal Antibody Polymer Reagent (ImmPRESS^®^ HRP Universal PLUS Polymer Kit, Peroxidase, Vector Laboratories). After washing in PBS, the reaction was visualized with ImmPACT DAB EqV Substrate (ImmPRESS^®^ HRP Universal PLUS Polymer Kit, Peroxidase, Vector Laboratories). After visualization slides were counterstained with hematoxilin (Harris modified Hematoxilin, Sigma, Merck, Darmstadt, Germany) and mounted in Histokitt (CarlRoth, GmbH, Karlsruhe, Germany) mounting medium and evaluated under an Olympus IX81 inverted microscope (Olympus, Hamburg, Germany). Micrographs were collected with CellSens Standard 1.5 software (Olympus, Hamburg, Germany). A positive reaction was described as yellow-to-brown pigmentation found in nuclei and cellular membranes. Each slide was evaluated by two independent observers.

### 4.3. Statistical Calculations

StatView 5.0 software (Carry, NC, USA) was used for all statistical analyses, including chi-square, odds ratios, and confidence intervals. Fisher’s PLSD test was used to determine immunohistochemical expression of LAG-3 and TIM-3 in ovarian cancer tissue samples in relation to the factors under analysis. A *p*-value < 0.05 was regarded as statistically significant. The group with expression intensity 0–1 and the group with expression intensity 2-4 were created for statistical calculations using Fisher’s PLSD test. Correlation analyses were performed using Spearman’s rank method.

## 5. Conclusions

Comparing the HGSOC group with the non-HGSOC group, both patients with lower and higher LAG-3 protein expression showed differences in grade and histological type. In addition, patients with higher LAG-3 protein expression showed differences in menopausal status, FIGO stage, and BMI, which was not observed in patients with lower expression. It was shown that both patients with lower and higher expression of TIM-3 protein showed a difference in grade, histological type, and FIGO stage.

It was also shown that LAG-3 could be a marker associated with BMI in the non-HGSOC group. It was found that TIM-3 may be a marker associated with age in a group of all ovarian cancers. LAG-3 expression is associated with TIM-3 expression in the total cohort and the HGSOC and non-HGSOC groups.

Although TIM-3 and LAG-3 are promising clinical markers, it also seems important to expand research in the use of these molecules in the treatment of ovarian cancer, as is carried out in breast cancer or melanoma. Thus, it is important to continue research not only on the clinical significance of these markers but also to consider them as potential therapeutic targets. While surgery and carboplatin–paclitaxel chemotherapy are the first-line treatments, TIM-3 and LAG-3 inhibitors, along with PD-1/PD-L1 and CTLA-4 inhibitors, could be considered as adjuvant treatments. They might also be taken into consideration in conjunction with bevacizumab and PARP inhibitors. This would require experimental models and clinical trials.

However, it should be noted that the results presented are preliminary and require validation in a larger cohort.

## Figures and Tables

**Table 1:** Clinical–demographical characteristics. * Fisher’s exact *p*-value; OR—odds ratio; CI—confidence interval; NS—non-significant.

Clinical–Demographical Characteristics	Total Cohort (*n* = 58)	Non-HGSOC (*n* = 26)	HGSOC (*n* = 32)	*p*-Value *	*OR (95%CI)*
**Median (IQR)**					
Age (years old)	63.0 (20)	51.0 (23)	63.5 (12.5)	*0.005*	
BMI (kg/m^2^)	24.7 (6.9)	25.4 (6.6)	23.4 (7.1)	*NS*	
**Number (%)**					
**LAG-3 protein expression intensity**					0.7 (0.3–2.1)
0-1	27 (46.6%)	11 (42.3%)	16 (50%)	*NS*	
2-4	31 (53.4%)	15 (57.7%)	16 (50%)		
**TIM-3 protein expression intensity**					0.5 (0.2–1.6)
0-1	18 (31.0%)	6 (23.1%)	12 (37.5%)	*NS*	
2-4	40 (69.0%)	20 (76.9%)	20 (62.5%)		
Age (years old)					
<65	33 (56.9%)	17 (65.4%)	16 (50%)	*NS*	
≥65	25 (43.1%)	9 (34.6%)	16 (50%)		
BMI					
<25	30 (51.7%)	11 (42.3%)	19 (59.4%)	*NS*	
≥25	28 (48.3%)	15 (57.7%)	13 (40.6%)		
Menopausal status					
Premenopausal	20 (34.5%)	14 (53.8%)	6 (18.7%)	*0.006*	
Postmenopausal	38 (65.5%)	12 (46.2%)	26 (81.3%)		
Grade					
High (G2 + G3)	40 (69.0%)	8 (30.8%)	32 (100%)	*0.0001*	
Low (G1)	18 (31.0%)	18 (69.2%)	0 (0%)		
Histological type					
Serous	44 (75.9%)	12 (46.2%)	32 (100%)	*0.0001*	
Non-serous	14 (24.1%)	14 (53.8%)	0 (0%)		
FIGO					
I–II	19 (32.8%)	15 (57.7%)	4 (12.5%)	*0.0002*	
II–IV	39 (67.2%)	11 (42.3%)	28 (87.5%)		

**Table 2:** Comparison of clinical characteristics in groups 0-1 of LAG-3 protein expression intensity between HGSOC and non-HGSOC. * Fisher’s exact *p*-value; NS—non-significant.

	Non-HGSOC (*n* = 11)	HGSOC (*n* = 16)	*p*-Value *
LAG-3 (0-1)			
Grade			
High	4 (36.4%)	16 (100%)	*0.0002*
Low	7 (63.6%)	0 (0%)	
Histological type			
serous	5 (45.5%)	16 (100%)	*0.0008*
Non-serous	6 (54.5%)	0 (0%)	
Menopausal status			
Premenopausal	5 (45.5%)	4 (25%)	*NS*
Postmenopausal	6 (54.5%)	12 (75%)	
FIGO			
I-II	6 (54.5%)	3 (18.8%)	*NS*
III-IV	5 (45.5%)	13 (81.2%)	
BMI			
<25	7 (63.6%)	9 (56.3%)	*NS*
>25	4 (36.4%)	7 (43.7%)	
Age			
<65	7 (63.6%)	9 (56.3%)	*NS*
>65	4 (36.4%)	7 (43.7%)	

**Table 3:** Comparison of clinical characteristics in groups 2-4 of LAG-3 protein expression intensity between HGSOC and non-HGSOC. * Fisher’s exact *p*-value; NS—non-significant.

	Non-HGSOC (*n* = 15)	HGSOC (*n* = 16)	*p*-Value *
LAG-3 (2-4)			
Grade			
High	4 (26.7%)	16 (100%)	*0.0001*
Low	11 (73.3%)	0 (0%)	
Histological type			
Serous	7 (46.7%)	16 (100%)	*0.0007*
Non-serous	8 (53.3%)	0 (0%)	
Menopausal status			
Premenopausal	9 (60%)	2 (12.5%)	*0.005*
Postmenopausal	6 (40%)	14 (87.5%)	
FIGO			
I–II	9 (60%)	1 (6.3%)	*0.001*
III–IV	6 (40%)	15 (93.7%)	
BMI			
<25	4 (26.7%)	10 (62.5%)	*0.04*
>25	11 (73.3%)	6 (37.5%)	
Age			
<65	10 (66.7%)	7 (43.8%)	*NS*
>65	5 (33.3%)	9 (56.2%)	

**Table 4:** Comparison of clinical characteristics in groups 0-1 of TIM-3 protein expression intensity between HGSOC and non-HGSOC. * Fisher’s exact *p*-value; NS—non-significant.

	Non-HGSOC (*n* = 6)	HGSOC (*n* = 12)	*p*-Value *
TIM-3 (0–1)			
Grade			
High	3 (50%)	12 (100%)	*0.007*
Low	3 (50%)	0 (0%)	
Histological type			
Serous	3 (50%)	12 (100%)	*0.007*
Non-serous	3 (50%)	0 (0%)	
Menopausal status			
Premenopausal	2 (33.3%)	1 (8.3%)	*NS*
Postmenopausal	4 (66.7%)	11 (91.7%)	
FIGO			
I–II	3 (50%)	1 (8.3%)	*0.04*
III-IV	3 (50%)	11 (91.7%)	
BMI			
<25	4 (66.7%)	8 (66.7%)	*NS*
>25	2 (33.3%)	4 (33.3%)	
Age			
<65	4 (66.7%)	6 (50%)	*NS*
>65	2 (33.3%)	6 (50%)	

**Table 5:** Comparison of clinical characteristics in the groups of 2-4 TIM-3 protein expression intensity between HGSOC and non-HGSOC. * Fisher’s exact *p*-value; NS—non-significant.

	Non-HGSOC (*n* = 20)	HGSOC (*n* = 20)	*p*-Value *
TIM-3 (2-4)			
Grade			
High	5 (25%)	20 (100%)	*0.0001*
Low	15 (75%)	0 (0%)	
Histological type			
Serous	9 (45%)	20 (100%)	*0.0001*
Non-serous	11 (55%)	0 (0%)	
Menopausal status			
Premenopausal	12 (60%)	15 (75%)	*NS*
Postmenopausal	8 (40%)	5 (25%)	
FIGO			
I–II	12 (60%)	3 (15%)	*0.003*
III–IV	8 (40%)	17 (85%)	
BMI			
<25	7 (35%)	11 (55%)	*NS*
>25	13 (65%)	9 (45%)	
Age			
<65	13 (65%)	10 (50%)	*NS*
>65	7 (35%)	10 (50%)	

**Table 6:** Spearman’s rank correlations for the total ovarian cancer cohort.

	Rho	*p*-Value
LAG-3, TIM-3	0.609	<0.0001
LAG-3, age	−0.068	0.6129
LAG-3, BMI	0.143	0.2864
TIM-3, age	−0.291	0.0262
TIM-3, BMI	0.063	0.6387

**Table 7:** Spearman’s rank correlations for the HGSOC.

	Rho	*p*-Value
LAG-3, TIM-3	0.535	0.0013
LAG-3, age	0.125	0.4995
LAG-3, BMI	−0.084	0.6515
TIM-3, age	−0.161	0.3828
TIM-3, BMI	−0.114	0.5387

**Table 8:** Spearman’s rank correlations for the non-HGSOC.

	Rho	*p*-Value
LAG-3, TIM-3	0.710	<0.0001
LAG-3, age	−0.164	0.4270
LAG-3, BMI	0.405	0.0394
TIM-3, age	−0.223	0.2766
TIM-3, BMI	0.239	0.2433

## Data Availability

The data presented in this study are available from the corresponding author, D.B., upon reasonable request.
